# Increased Default Mode Network Connectivity in Obsessive–Compulsive Disorder During Reward Processing

**DOI:** 10.3389/fpsyt.2018.00254

**Published:** 2018-06-12

**Authors:** Kathrin Koch, Tim J. Reeß, Oana G. Rus, Deniz A. Gürsel, Gerd Wagner, Götz Berberich, Claus Zimmer

**Affiliations:** ^1^Department of Neuroradiology, Klinikum rechts der Isar, Technische Universität München, Munich, Germany; ^2^TUM-Neuroimaging Center of Klinikum rechts der Isar (TUM-NIC), Technische Universität München, Munich, Germany; ^3^Graduate School of Systemic Neurosciences, Ludwig-Maximilians-Universität, Munich, Germany; ^4^Department of Neuroradiology, University of Zurich, Zurich, Switzerland; ^5^Department of Psychiatry and Psychotherapy, Jena University Hospital, Jena, Germany; ^6^Windach Institute and Hospital of Neurobehavioural Research and Therapy, Windach, Germany

**Keywords:** OCD, reward, reinforcement, connectivity, DMN

## Abstract

**Objective:** Obsessive-compulsive disorder (OCD) is characterized by anxiety-provoking, obsessive thoughts (i.e., obsessions) which patients react to with compulsive behaviors (i.e., compulsions). Due to the transient feeling of relief following the reduction of obsession-induced anxiety, compulsions are often described as relieving or even rewarding. Several studies investigated functional activation during reward processing in OCD, but findings are heterogeneous up to now and little is known about potential alterations in functional connectivity.

**Method:** Against this background we studied OCD patients (*n* = 44) and healthy controls (*n* = 37) during the receipt of monetary reward by assessing both activation and functional connectivity.

**Results:** Patients showed a decreased activation in several frontal regions and the posterior cingulate (PCC, BA31) together with a stronger connectivity between the PCC and the vmPFC (BA10).

**Conclusion:** Present findings demonstrate an increased connectivity in patients within major nodes of the default mode network (DMN)—a network known to be involved in the evaluation of internal mental states. These results may indicate an increased activity of internal, self-related processing at the expense of a normal responsiveness toward external rewards and incentives. This, in turn, may explain the constant urge for additional reinforcement and patients' inability to inhibit their compulsive behaviors.

## Introduction

Obsessive-compulsive disorder (OCD) is characterized by anxiety-provoking, involuntary, obsessive thoughts which patients react to with repetitive, compulsive behavior patterns to counteract anxiety. These reactive compulsive behavior patterns are often perceived by the patients as highly remunerating because of their rewarding impact after reducing obsession-related anxiety. Over time these behavior patterns may take on an addictive character and lead to an altered processing of natural rewards in association with an altered functioning of the reward system. Hence, the reward system may become hyposensitive to dopamine as a homeostatic response to continuous activation—a mechanism which has been demonstrated in several neuropsychiatric conditions (1). This continuous activation may go along with a tonic increase of dopamine in the synaptic cleft. Some of the dopamine is brought back into the presynaptic cell by reuptake, whereas the rest continues to activate the postsynaptic cell. The abundance of dopamine may then trigger the cell to be less receptive to new dopamine via the dopamine receptor D2. Thus, the tonic activity of the reward system may decrease and an increasing amount of stimulation may be necessary to reach the normal level of activity (1). On the basis of these somewhat speculative considerations a significantly decreased responsiveness of areas within the reward system has been hypothesized as one psychopathologically relevant mechanism in OCD (2).

A majority of the studies on reward-related brain activation in OCD support this hypothesis. They report altered activation—mainly in terms of a decreased activation in OCD patients compared to healthy subjects—in frontal and striatal regions as well as in the hippocampus-amygdala complex (2–6).

Apart from these studies on altered functional activation in association with the processing of reward in OCD, first attempts have been made to bring more insight into potential network disturbances by investigating alterations in functional connectivity.

A study assessing functional connectivity during resting state and during reward processing reported, as a major result, a decreased functional connectivity between the nucleus accumbens (Nacc) and the amygdala in OCD patients compared to healthy controls during incentive processing in the monetary incentive delay task (7). As opposed to these results findings by Admon et al. (8) indicated altered functional connectivity in OCD patients compared to healthy controls not in-between these regions but of the amygdala and Nacc to two frontal regions, the orbito-frontal cortex (OFC) and the dorsal anterior cingulate cortex (dACC), respectively.

Viewed together, findings on functional activation and connectivity in OCD in the context of reward processing point to alterations in mainly frontal and striatal regions despite some result heterogeneity. This heterogeneity might be due to methodological differences (i.e., reward anticipation vs. reward receipt, differential modeling of reward processing, differential instrumentalization of reward/positive reinforcement) and/or sample characteristics (i.e., medicated vs. unmedicated patients, patients with vs. patients without comorbidities, differences regarding symptom severity, symptom profiles and duration of illness). Apart from that, little is known about potentially relevant alterations in functional connectivity in the context of reward processing in OCD up to now.

### Aims of the study

In the present study, we sought to explore the neural basis of reward system function in OCD by comparing functional activation between OCD patients and healthy control subjects during reward processing using a robust task based on monetary incentives. In addition, we aimed at investigating whether potential alterations in functional activation are directly associated with potential alterations in functional connectivity which would indicate network disturbances as an underlying mechanism.

## Materials and methods

The study included a right-handed sample of 44 OCD patients and 37 healthy controls matched for age and gender (Table 1). Handedness was assessed using Annett's questionnaire (9). Exclusion criteria for both groups were a history of clinically important head injuries, seizures or neurological diseases. Healthy controls with a history of psychiatric illness were not included in the study.

**Table 1:** Demographic and clinical data.

	**OCD (*N* = 44)**	**Controls (*N* = 37)**	**Group difference**
**Characteristic**	**Mean (SD)**	**Mean (SD)**	***p*-value**
Sex, male: female	17:27	15:22	*χ*^2^ = 0.03, *p* = 0.86
Age, Years	32.7 (9.3)	32.0 (8.0)	*t* = −0.12, *p* = 0.90
Medication, yes/no	32/12		NA
Medication type	20 SSRI		
	4 SNRI		
	4 TrA		
	1 Benzo		
	1 Atypic		
	2 no info		
Comorbidities Present/not present	26/18		NA
Comorbidity type	16 depression		
	1 anxiety disorders		
	5 depression & anxiety disorder		
	2 personality disorder		
	1 impulse control		
	disorder-not otherwise specified		
Age at onset	16.23 (6.6)		NA
YBOCS total	20.0 (6.8)		NA
-Obsessions	10.5 (3.7)		
-Compulsions	9.4 (4.2)		

*NA, not applicable; SD, standard deviation; SSRI, selective serotonin reuptake inhibitor; SNRI, serotonin-norepinephrine reuptake inhibitor; TrA, tricyclic antidepressant; Benzo, benzodiazepine; Atypic, Atypical Antipsychotic*.

The patients were recruited from the in-patient hospital ward specialized on OCD of the Windach Institute and Hospital of Neurobehavioural Research and Therapy, Germany. This ward has a standardized admission process including psychopathological screenings and a disorder history assessment performed by an experienced psychiatrist. The final diagnosis of OCD was based on a structured DSM-IV interview. Prior to the scanning session, we additionally assessed the severity of symptoms and the characteristics of the disorder using the Yale-Brown Obsessive Compulsive Scale (Y-BOCS) (10). Patients with medication and comorbidities were also included, provided that OCD was the primary diagnosis.

All participants gave written informed consent to the study protocol. The protocol is in accordance with the Declaration of Helsinki and was approved by the Ethics Committee of the Technische Universität München, Medical School.

### Experimental design

Using the Presentation software package (Neurobehavioral Systems Inc., USA) stimuli were projected onto a transparent screen inside the scanner tunnel which could be viewed by the subject through a mirror system mounted on top of the MRI head coil. The subjects' responses were recorded using an MRI-compatible fiber optic response device (Lightwave Medical Industries, Canada) with a four button keypad for the right hand. To investigate reward-related activation, we used a paradigm based on monetary reward (11–13). Participants were informed that they will be presented a geometrical figure (i.e. cross, half-moon, triangle, or pentagon) and asked to guess whether the figure predicted a number higher or lower than the number five. Each figure predicted the respective number with a probability of either 50 or 100%. These probability conditions (50 and 100%) were chosen to investigate reward processing after maximum and minimum decision uncertainty. As such the task has a probabilistic character and is adequate for investigating reward processing.

Each correct guess was followed by a monetary reward (+0.50 €) whereas each wrong guess was followed by a punishment (−0.50 €). Participants were instructed that the figure predicted the respective number with a certain probability but were not informed about the predictive probabilities of the respective figures. The whole paradigm consisted of a series of 64 interleaved trials in which the 32 trials for each probability condition were distributed across the whole task sequence for each probability condition. Each trial started with the presentation of the probability condition-specific figure which was shown for 1.5 s. After an inter-stimulus interval lasting 4.5 s, a question mark was presented for 2.5 s during which participants had to answer by button press. After another interstimulus interval of 4.5 s, the correct solution followed by the indication of a reward or punishment appeared for 2.5 s.

Each trial ended with an inter-trial interval lasting 3.5 s. In addition, we introduced a temporal jitter by varying the second inter-stimulus interval between 4.5 and 5.5 s in order to increase sensitivity. Participants were compensated according to their performance, although a minimum of € 20 was guaranteed for volunteering.

### fMRI data acquisition and processing

Functional MR images were acquired in a 3 Tesla whole body MR scanner (Achieva, Philips, The Netherlands) using an 8-channel phased-array head coil. FMRI data consisted of 644 volume scans, which were collected by using a gradient echo EPI sequence (TE = 30 ms, TR = 2,000 ms, flip angle = 90°, FoV = 192 × 192 × 122 mm, matrix = 64 × 64, 37 slices, slice thickness = 3 mm, and 0 mm interslice gap).

High-resolution T1-weighted anatomical scans were obtained using a magnetization-prepared rapid acquisition gradient echo (MPRAGE) sequence with the following scanning parameters: repetition time (TR) = 9 ms, echo time (TE) = 4 ms, inversion time = 1,000 ms, flip angle = 8°, matrix size = 240 × 240 mm^2^, number of slices = 170, acceleration factor (SENSE) = 2 with an isotropic resolution of 1 × 1 × 1 mm^3^.

Processing of the images and statistical analysis were performed with SPM 12 (http://www.fil.ion.ucl.ac.uk/spm; Wellcome Trust Centre for Neuroimaging, University College London, UK) in MATLAB 8.2.0 (R2015a, Mathworks, CA). Functional data were corrected for differences in time of acquisition by sinc interpolation, realigned to the first image of every session and linearly and non-linearly normalized to the Montreal Neurological Institute (MNI, Montreal, Canada) reference brain (MNI 152). Data were spatially smoothed with a Gaussian kernel (8 mm, full-width at half-maximum) and high-pass filtered with a 128 s cut-off. All data were inspected for movement artifacts. Subjects with movement parameters exceeding 3 mm translation on the x-, y-, or z-axis or 3° rotation were excluded. In addition, individual movement parameters entered analyses as covariates of no interest.

On the first level, brain activations were then analyzed voxel-wise to calculate statistical parametric maps of *t*-statistics for the 50% probability condition (i.e., activation during presentation of or responding to triangles and pentagons), the 100% probability condition (i.e., activation during presentation of or responding to half-moons and crosses), positive feedback/reward (i.e., activation during presentation of positive feedback and monetary win) and negative feedback/punishment (i.e., activation during presentation of negative feedback and monetary loss).

Blood oxygenation level dependent (BOLD) signal changes for the different conditions were modeled as a covariate of variable length boxcar functions and convolved with a canonical hemodynamic response function (HRF). These HRFs were then used as individual regressors within the general linear model (GLM). One-sample *t*-tests were performed on the second level to analyze reward-related activation (i.e., activation during presentation of positive feedback/monetary reward). A one-way ANCOVA with age and gender as covariates of no interest was used to compare reward-related activation between the groups. In addition, to investigate a potential effect of medication, a two-sample *t*-test was performed to compare reward-related activation between medicated (*n* = 32) and umedicated (*n* = 12) patients. The results were corrected for multiple comparisons by using the threshold free cluster enhancement (TFCE) approach as implemented in SPM (14), with *p* < 0.05 FWE corrected and 5,000 permutations.

**Figure 1:**
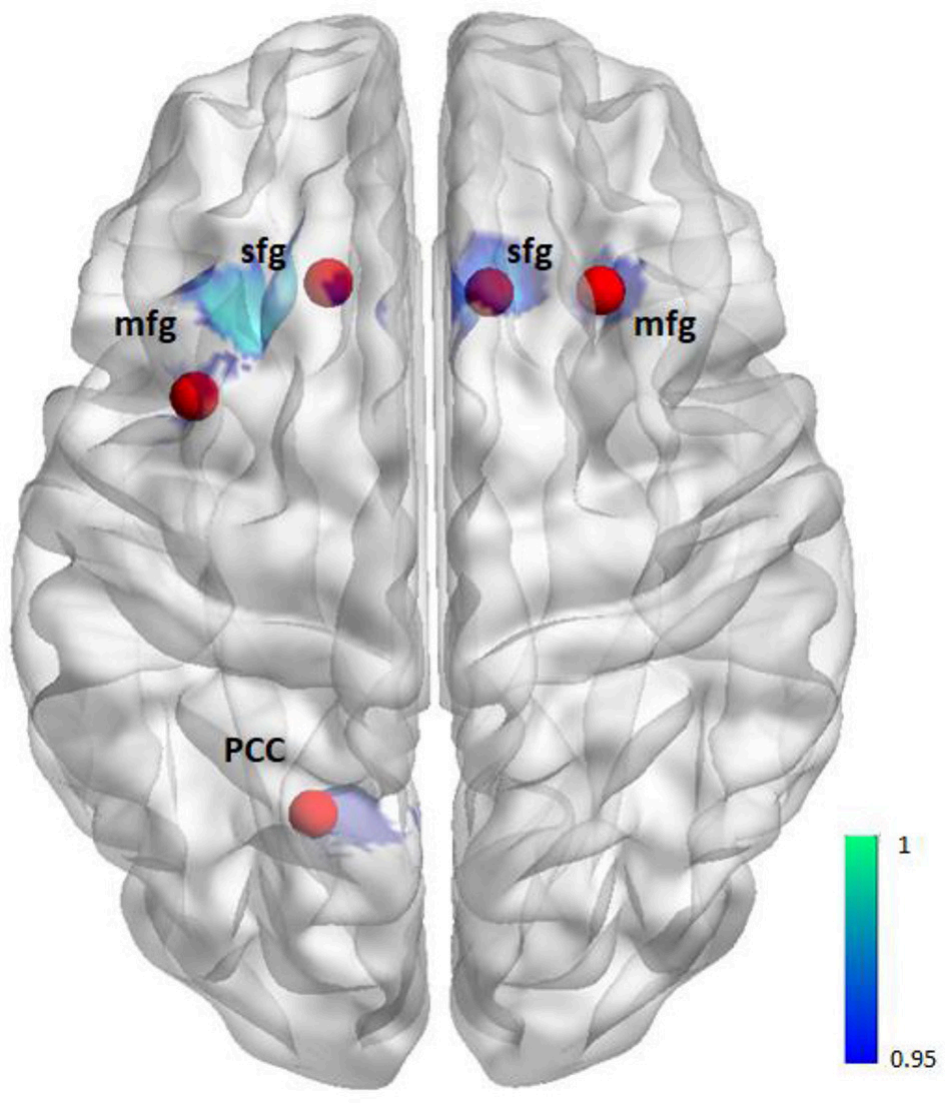
Illustration of seeds for the seed-to-voxel functional connectivity analysis. Seeds (red) are based on the activation (blue) from the group comparison (i.e., 6 mm sphere centered on the maximum activated voxel for the contrast controls > patients positive feedback/reward, see also Table 2).

To investigate whether altered reward-related functional activation was associated with altered functional connectivity we used the CONN toolbox (15) and the seed-to-voxel approach to perform post-hoc connectivity analyses for those clusters showing a significantly altered activation in patients compared to healthy controls (Figure 1). Clusters with a spatial extent of less than *k* = 20 voxels were not taken into consideration.

These clusters showing a significantly altered activation in patients compared to controls (i.e., 6 mm sphere centered on the maximum activated voxel) determined the seed regions. The mask including all seeds was generated using the WFU PickAtlas (http://fmri.wfubmc.edu/software/pickatlas). BOLD time courses were extracted from the seed regions and Pearson's correlation coefficients were computed between each seed time course and every other voxel in the brain to investigate reward-related functional connectivity between the seed region and the rest of the brain (i.e., we chose the option “weighted GLM” which means that the seed-to-voxel bivariate correlation measure obtained for the task condition represents the connectivity during that task condition).

Correlation coefficients were converted using Fisher's transform to normally distributed scores for second level analysis to compare groups. Again, second level maps were corrected for multiple comparisons by using the threshold free cluster enhancement (TFCE) approach as implemented in SPM with *p* < 0.05 FWE corrected and 5,000 permutations.

Realignment parameters, BOLD signal from the white matter and cerebrospinal fluid masks and task effects were entered as covariates of no interest on the first level and age as well as gender were entered as covariates of no interest on the second level.

Finally, to *post-hoc* investigate whether altered functional connectivity between our seed region (i.e., left PCC/precuneus, BA31) and the clusters found to be altered in connectivity in patients (i.e., left VMPFC/BA10 and right PCC; see also Table 2) was related to symptom severity we correlated parameter estimates extracted from these clusters (i.e., 6 mm sphere around maximum activated voxel at *x* = −6, *y* = 60, *z* = −6 and *x* = 4, *y* = −52, *z* = 22) with Y-BOCS total scores.

**Table 2:** MNI coordinates of activation maxima for the group comparison positive feedback/reward (controls > patients at *p* < 0.05 corrected) (upper part).

	**Side**	***k***	***p*_corr_**	***x, y, z***
**BRAIN REGIONS WHOLE BRAIN ACTIVATION**				
Superior frontal gyrus, BA8	L	352	0.031	−16, 26, 48
Superior frontal gyrus, BA6	R	148	0.036	10, 24, 52
Middle frontal gyrus, BA6	L	34	0.048	−38, 8, 54
Middle frontal gyrus, BA8	R	28	0.046	28, 24, 46
PCC, precuneus, BA31	L	43	0.048	−20, −60, 24
Precentral gyrus, BA6	L	18	0.046	−40, −4, 30
ACC, BA32	L	1	0.047	−10, 20, 40
**BRAIN REGIONS PCC CONNECTIVITY**				
Superior frontal gyrus, medial frontal gyrus, BA10	L	1,554	0.004	−6, 60, −6
PCC, BA31	R	263	0.011	4, −52, 22
Medial frontal gyrus, BA10	L	1	0.031	−14, 58, 4
ACC, BA32	R	1	0.041	6, 46, 2
Medial frontal gyrus, BA10	R	1	0.047	8, 54, 12

*MNI coordinates of activation maxima for increased l. PCC (6 mm sphere around maximum activated voxel at x = −20 y = −60 z = 24) connectivity in patients compared to controls for positive feedback/reward (patients > controls at p < 0.05 corrected, lower part). L/R, left/right side; k, number of voxels in cluster; p_corr_, corrected p-value; x, y, z, MNI coordinates in mm*.

## Results

**Figure 2:**
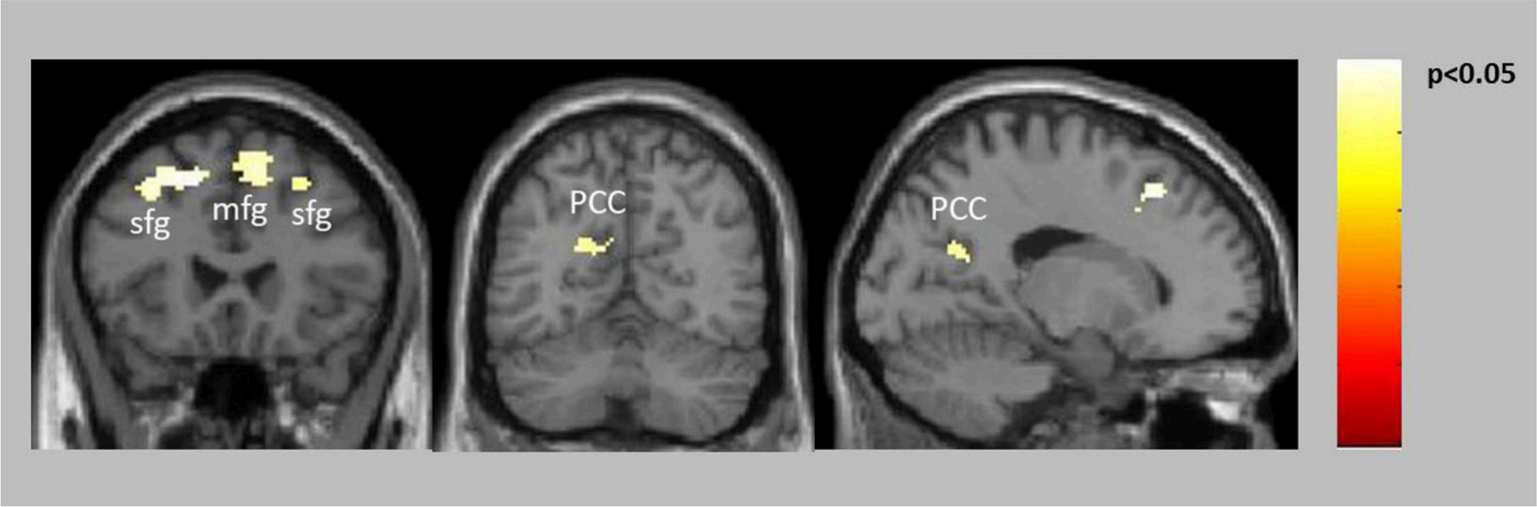
Decreased activation in patients compared to controls for positive feedback/reward (see also Table 2).

Both groups showed significant reward-related activation in an extended network containing mainly medial and lateral frontal, parietal and striatal regions (see Supplementary Figure 1). The two-sample *t*-test comparing punishment-related activation between the groups yielded no activation differences. The ANCOVA comparing reward-related activation between the groups yielded no activation increases in patients compared to healthy controls but significantly decreased activation in patients compared to controls in the frontal cortex bilaterally (BA6, BA8) and the posterior cingulate extending into the left precuneus (Table 2, Figure 2). The two-sample *t*-test comparing reward-related activation between medicated and unmedicated patients did not yield any significant results.

**Figure 3:**
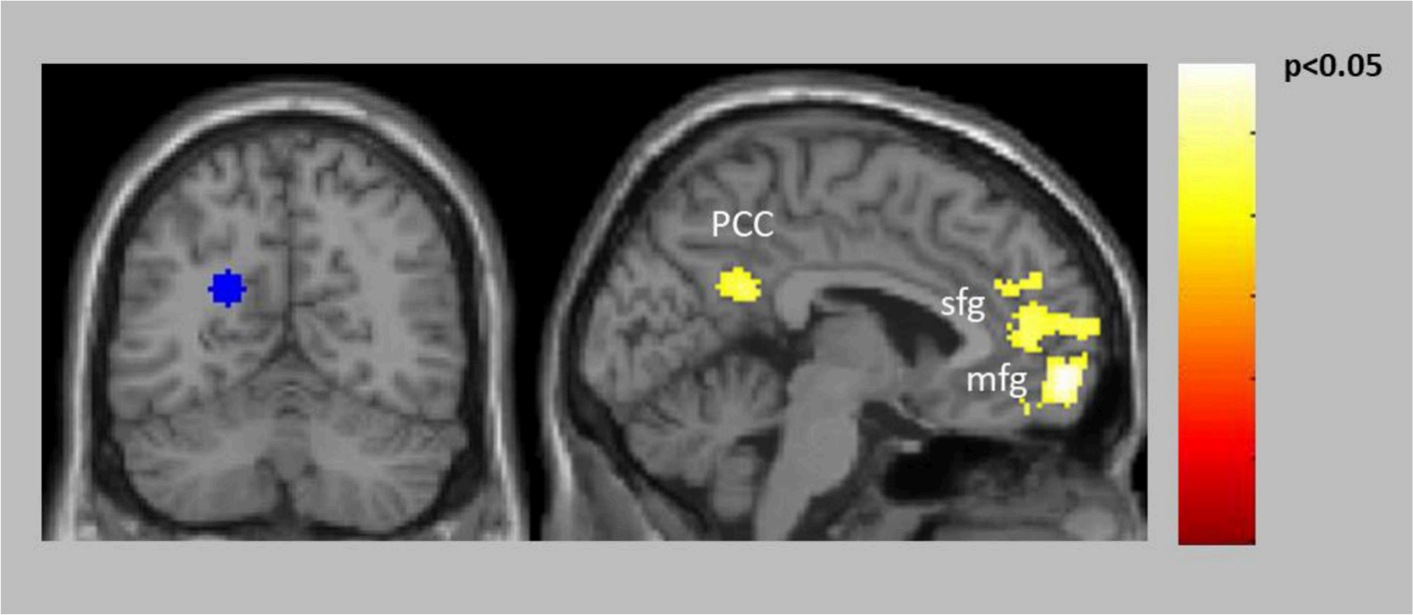
Increased connectivity in patients compared to controls between the left PCC/precuneus (BA31) seed (blue) and left vmPFC (BA10) as well as right PCC (BA31).

The connectivity analyses exploring potential alterations in reward-related connectivity in patients compared to healthy controls yielded no significantly decreased connectivity in patients but a significantly increased connectivity between the left PCC/precuneus (BA31, seed with maximum activated voxel at *x* = −20, *y* = −60, *z* = 24) and the left vmPFC (BA10) and the right PCC (BA31) (Table 2, Figure 3, for within-group results see Supplementary Figure 2).

Connectivity analyses for the other four seed regions (for center coordinates please refer to Table 2) yielded no significant differences between the groups. The correlation between Y-BOCS total and connectivity between left PCC and left vmPFC/BA10 was not significant (*r* = 0.08, *p* = 0.6). The correlation between Y-BOCS total and connectivity between left PCC and right PCC was also not significant (*r* = −0.02, *p* = 0.9).

## Discussion

In the present study we investigated potential alterations in functional activation and connectivity in patients with OCD in the context of reward processing. We found that compared to healthy controls patients with OCD exhibited a decreased functional activation in frontal regions and the posterior cingulate (PCC) during the processing of positive feedback and monetary reward. Moreover, patients showed an increased functional connectivity between the left PCC and the right ventromedial prefrontal cortex (vmPFC, BA10) as well as between left and right PCC.

Our findings are partly in line with previous studies which reported a decreased functional activation in medial frontal regions in patients with OCD compared to healthy participants in association with the processing of reward (4, 6). However, some of these previous studies also demonstrated a blunted response in OCD patients within the more characteristic components of the reward system, such as in dorsal and ventral striatal regions (2, 3, 5). Interestingly, in the present study we found unimpaired responsiveness of these characteristic regions in patients in association with the processing of positive feedback and monetary reward (see Supplementary Figure 1). Instead, patients showed a significantly decreased activation in the PCC (area 31) including anterior parts of the precuneus. This obvious inconsistency between the present findings and preceding results (2–5, 7, 16, 17) confirms once more the general result heterogeneity with respect to reward processing in OCD. These inconsistencies might be due to methodological differences and/or sample characteristics. Namely, some of the studies investigated unmedicated patients (3, 5) or assessed functional activation during the anticipation of reward (2, 4, 16). There is a strong reason to assume that the cognitive and affective processes characterizing the mere mental visualization or expectation of a rewarding stimulus differ significantly from those processes accompanying the actual receipt of a positive feedback or reward (18). Upon the receipt of a rewarding stimulus, our reward system responds with an increased activity. This increased activity assigns the stimulus its transiently rewarding character. The present findings indicate that this basic reward mechanism might be intact in patients with OCD (see also Supplementary Figure 1 illustrating activation of the characteristic reward network in both groups). The decreased activation in the posterior part of the cingulate cortex suggests that it is not so much the general functionality of the reward system that is altered in OCD but rather certain secondary processes accompanying the processing of a rewarding stimulus. Anatomically, the posterior cingulate cortex is situated in the medial part of the inferior parietal lobe and lies within the posteromedial cortex, which also includes the precuneus and retrosplenial cortex (19). It represents part of the limbic system that is often described as the “emotional brain” (20). Functionally, the PCC is critically involved in the regulation of arousal and attention (21–23) and, as formulated in a recent review article (23), might control the balance between internally and externally focused thought (24). Activity in the PCC has been shown to vary with arousal state and to critically determine conscious awareness (19). Accordingly, in low states of arousal and awareness or anesthesia (25), and in the vegetative state (26–29) PCC activity has been demonstrated to be significantly reduced whereas it significantly increases as soon as consciousness is regained and arousal increases. Likewise, PCC activity has been shown to increase during attentional biasing to targets that are of high motivational value (30). Studies in primates found an increased neuronal activity in the PCC when risky choices were made and PCC activation was better predicted by the subjective motivational value and arousal caused by a chosen target than by its actual value (31). In this light, Leech and Sharp (23) assume that the PCC may be critically involved in maintaining a certain level of responsiveness or arousal (21, 32, 33), or in signaling behaviourally relevant changes in the environment (34).

Hence, the decreased PCC activity in patients during the reward outcome may, first, indicate a lower level of arousal and responsivity to positive feedback and reward. Thus, present findings suggest that although patients' reward system may respond “normally” toward reward, secondary processes such as physical arousal and physical-affective responsiveness to reward may be impaired.

Of note, two systematic reviews on gray and white matter changes in OCD (35, 36) pointed to structural alterations in the PCC concluding that—in addition to the regions “classically” implicated in OCD pathogenesis—posterior brain regions may be of greater psychopathological relevance for the disorder than previously thought. Considering that the OFC, one major node within the classical cortico-striato-thalamo-cortical (CSTC) circuit, targets not only the ventromedial head of caudate and anterior parts of the cingulate, but also the PCC (37), this assumption is plausible also from an anatomical perspective. Hence, interpreted against the background of the classical CSTC model of OCD, present findings suggest that OCD patients may suffer from an increased or disinhibited responsiveness to symptom-relevant stimuli (due to an imbalance between direct and indirect CSTC pathways in favor of the direct excitatory pathway) going along with a blunted responsiveness toward other, usually rewarding, stimuli (due to a decreased PCC activity).

Second, if we assume that the decreased PCC activity in patients might indicate an impaired balance between internally and externally focused thought this might, in turn, explain the relative increase in connectivity with the vmPFC/rostral medial prefrontal cortex (BA10) which we found in patients with OCD compared to controls. The vmPFC and the PCC are functionally connected (38) and in interaction both regions constitute major parts of the default mode network (DMN). The DMN is an intrinsic brain network which has mostly been shown to be active through resting state studies when a person is not focused on the outside world, but is “mind-wandering” or thinking about himself (39). It has been found to show both hypo- and hyper-connectivity in patients with OCD in the resting state (40–42). Of note, a recent, comprehensive meta-analysis of resting state functional connectivity in OCD showed a dysconnectivity within the DMN peaking in the ACC and the vmPFC (43). Although the vmPFC as major part of the DMN has been found to be involved in a wide variety of tasks (44), it is assumed to be predominantly involved in processes related to mentalizing [i.e., attending to one's own emotions and mental states (45)]. This has also been shown by a large meta-analysis based on 104 functional imaging studies which came to the conclusion that attending to one's own inner states was predominantly associated with activation in the rostral prefrontal cortex (45). In other terms, or as Christoff and Gabrieli (46) put it, the vmPFC might be predominantly responsible “for the explicit processing of internal mental states and events—or introspective evaluation of one's own thoughts and feelings.” Accordingly, vmPFC activity has been observed in 13 out of 15 studies involving episodic memory tasks that require “evaluation of self-generated material” (44). Against this background, the increased connectivity between PCC and vmPFC (BA10) or within major nodes of the DMN might indicate an impaired balance between internally and externally focused thought reflecting a shift toward internally focused thought in patients with OCD.

Studies also reported that although the DMN is active during rest, certain parts of it such as vmPFC, ACC, precuneus and PCC are deactivated during performing various cognitive tasks (47, 48). Failure to deactivate the DMN or reduced deactivation within the structures of the DMN are reported in numerous psychiatric disorders such as autism, schizophrenia and depression (47–50). Moreover, reduced deactivation during task in patients with depression is associated with negative rumination (51). Such a mechanism of suppressing the DMN might play a role in changing the attention from internally directed thought to goal directed or task related behavior. Impairments in this mechanism, as seen in OCD patients in the current study, could represent a focus on the negative self-reference and inability to suppress intrusive thoughts. It should be noted, however, that further studies investigating the neural mechanisms underlying both the processing of external reinforcement and the processing of internal states in patients with OCD are needed to substantiate this provisional conclusion.

## Summary and limitations

In the present study, we found that OCD patients were characterized by a decreased activation in the PCC, potentially reflecting a blunted responsiveness to external reward, in association with an increased connectivity to the vmPFC which is strongly involved in the evaluation of internal processes. Thus, these alterations in activation and connectivity might indicate a shift of attentional focus away from external incentives toward internal processes in OCD. This, in turn, may lead to a decreased responsiveness toward external stimuli or incentives from the outer world. Clinically, this may be one mechanism underlying the constant and prevailing yearning for additional rewarding stimulation reflected in the inability to suppress or cease compulsive behaviors. It should be noted, however, that—somewhat in contradiction to this assumption—we found no association between PCC—vmPFC connectivity and symptom severity.

As a limiting factor it has to be noted that a majority of the patients were medicated and almost fifty percent suffered from a comorbid depression at the time of measurement. Although both factors may have influenced the results, the present patient sample can be regarded as representative of the OCD patient population as major depression predominantly constitutes a frequent comorbidity in OCD. As another limitation it should be mentioned that the present patient sample constitutes a mixed group of pediatric and non-pediatric onset OCD patients. Given the results of a recent meta-analysis (52) indicating that childhood-onset OCD may, in fact, be a subtype of the disorder the inclusion of both pediatric and non-pediatric onset patients may have confounded the results to some degree. Our assumption that secondary processes such as physical arousal and physical-affective responsiveness to reward may be impaired in OCD is speculative considering that we did not assess additional parameters such as skin conductance response (SCR). We also did not use any questionnaires to assess patients' affective responsiveness. Future studies should take these aspects into account.

## Author contributions

KK and GW designed the study and wrote the protocol. KK, TR, and OR managed the literature searches and analyses. KK and TR undertook the statistical analysis, and KK wrote the first draft of the manuscript. TR and OR recruited and screened the patients. CZ and GB acquired the financial resources and CZ, GB, and DG added additional aspects to the final manuscript. All authors have approved the final article.

### Conflict of interest statement

The authors declare that the research was conducted in the absence of any commercial or financial relationships that could be construed as a potential conflict of interest.
