# Supplementary figures and images for: Increased Default Mode Network Connectivity in Obsessive–Compulsive Disorder During Reward Processing
Source: Front Psychiatry. 2018 Jun 12;9:254. doi: 10.3389/fpsyt.2018.00254 (PMC6008536; doi:10.3389/fpsyt.2018.00254)

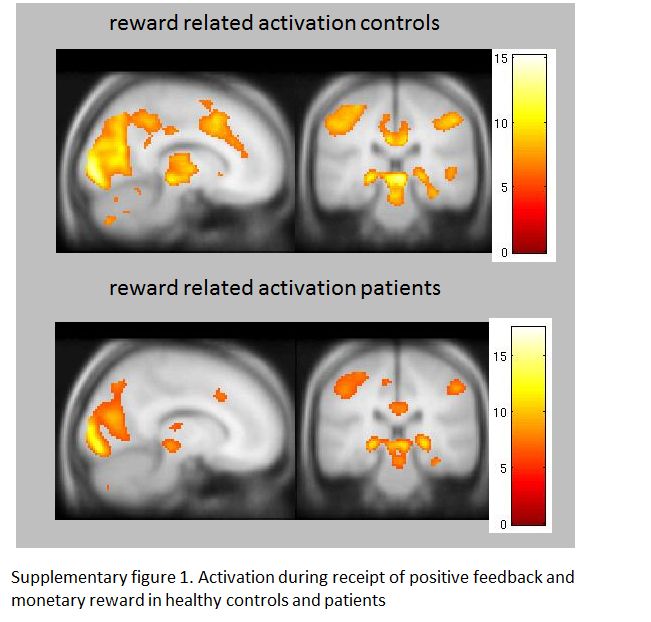

Supplement: Supplementary file 1 [file Image_1.JPEG]

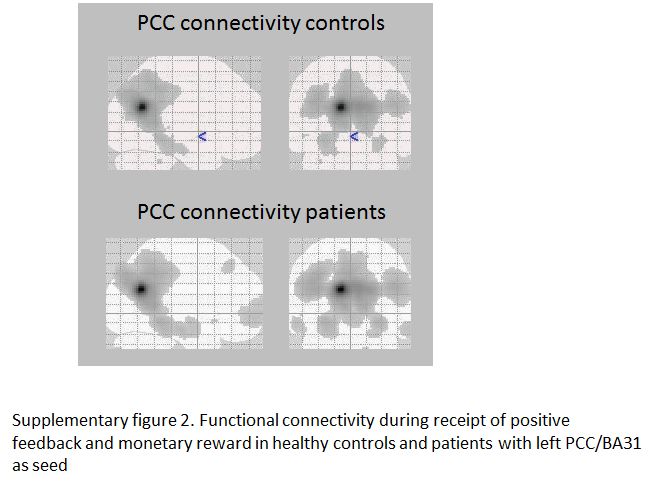

Supplement: Supplementary file 2 [file Image_2.JPEG]
